# The relationship between exercise intention and behavior of Chinese college students: A moderated mediation model

**DOI:** 10.3389/fpsyg.2022.1006007

**Published:** 2022-11-08

**Authors:** Lianghao Zhu, Junli Hou, Bojun Zhou, Xi Xiao, Jingqiang Wang

**Affiliations:** ^1^School of Physical Education, Hubei Business College, Wuhan, China; ^2^School of Physical Education, Sichuan Institute of Industrial Technology, Deyang, China

**Keywords:** exercise, intention-behavior gap, action planning, habit, dual process

## Abstract

Inconsistency between intention and behavior is very common in daily life. This study explored the intention-behavior relationship in exercise, focusing on the mediating effect of action planning and the moderating effects of habit strength and gender. For the purpose of providing theoretical reference for the implementation of intervention strategies in the volitional phase, a total of 489 college students (M-age = 20.61, 57.46% female) from Hubei Province, China, were recruited to complete the questionnaire at two time points. The findings showed that exercise intention could positively predict exercise behavior *via* the mediating effect of action planning, with the mediating effect accounting for 48.52% of the total effect. The predictive effect of action planning × habit strength interaction on exercise behavior was statistically significant. As individuals’ levels of habitual strength increased, so did the relationship between action plans and exercise behavior. The action planning-exercise behavior relationship was stronger in males than in females. In summary, action planning is a very important predictor of the post-intentional phase and has many advantages. For individuals whose exercise has become habitualized, forming a plan is not counterproductive and can still promote more exercise rather than in a mutually compensating manner.

## Introduction

It has long been accepted among Chinese college students that more exercise is beneficial to their health, and they frequently express favorable intentions toward exercise (Liang, 2015). The challenging part, however, is figuring out how to keep doing what one wants to do and successfully convert intention to action. Intention is an indication of a person’s readiness to perform a given behavior (Ajzen and Madden, 1986). It is considered as a proximal determinant of behavior (Ajzen, 1991). Previous research found a 46% disparity between intention and behavior in the area of physical activity, and this discrepancy was mostly ascribed to individuals who did not follow through on their intentions (Rhodes and de Bruijn, 2013). Ping (2022) indicated a moderate positive correlation between exercise intention and exercise behavior. Nonetheless, adopting the longitudinal designs with a lengthy assessment interval, the consistency of exercise intention and behavior was low between middle-aged and sub-healthy individuals, implying an intention-behavior gap may exist.

As the classical social cognitive theory suggests, exercise intention is the most direct and important predictor of individual exercise behavior (Blue, 1995), but the explanatory power of the former for the latter is weak in the volitional phase. Currently, it has been demonstrated that aspects such as executive function (Frye and Shapiro, 2021), self-efficacy (Isa et al., 2019; Divine et al., 2021), planning (Lange et al., 2018), and action control (Monge-Rojas et al., 2021) can provide some additional insights. However, while these studies mostly emphasized the importance of conscious regulatory processes, they neglected the implicit effects generated by automatic associations. Thus, this research focused on the formation mechanism of exercise intention to exercise behavior among Chinese college students and combined conscious and non-conscious regulatory processes for analysis, aiming at providing a theoretical reference for the implementation of intervention strategies in the post-intentional phase.

Action planning is a substructure of planning. It is a psychological simulation that associates goal-directed behaviors with specific contextual cues by defining when, where, and how to act (Sniehotta et al., 2005). In the Health Action Process Approach (Schwarzer, 2008), action planning is established as a mediating factor between intention and behavior. Not only is it an extension of intention, but it is also more likely and efficient than intention to trigger actual behavior when contextual cues are more adequate. In recent years, the interaction effects of action planning and other predictors have received much attention. Caudroit et al. (2014) discovered that action planning only worked as a moderator for people who had higher levels of coping planning. Self-efficacy was found to moderate the first and second halves of the action planning mediating process by Barz et al. (2016). This implies that the action planning mediates the predictive effect of exercise intention on exercise behavior may be influenced by a number of potential variables. Therefore, a foundational hypothesis 1 is proposed here: *Action planning mediates the predictive effect of exercise intention on exercise behavior of college students.*

According to contemporary theory and research, a habit is a behavior or behavioral tendency that is carried out automatically in response to a certain set of linked circumstances or contextual cues (Wood, 2017; Hagger, 2018). Once exercise turns into a habit, it will seem less deliberate to perform (Verplanken and Melkevik, 2008). It becomes a fast, automatic, and non-conscious process, similar to the description of “*system 1*” in Dual-Process Theories (Evans and Stanovich, 2013). On the one hand, previous research demonstrated that higher than average levels of intention predict behavior when individual habit strength levels are low (Di Maio et al., 2021). de Bruijn and Rhodes (2011) found the predictive effect of intention on behavior was roughly three times larger when habit strength was low compared to high levels of habit strength. On the other hand, some research pointed out that for high-intensity physical activity, individuals with high levels of habit strength showed a tighter connection between physical activity intention and behavior (Rhodes and de Bruijn, 2010). An identical conclusion was also reached by de Bruijn et al. (2012). Whether habit strength weakens or enhances the influence of exercise intention on exercise behavior is disputed. To be further verified, the following hypothesis 2 is proposed: *Habit strength moderates the predictive effect of exercise intention on the exercise behavior of college students.*

An increasing number of studies have confirmed the perspective that physical activity is the consequence of both conscious and unconscious processes (Rebar et al., 2016). The habit theory suggests that planning, a self-regulatory strategy frequently employed during habit formation, should have a direction that is compatible with the habit strength (Lally and Gardner, 2013). However, Maher and Conroy’s (2015) 7-day intervention study found that the conscious-control behavior of creating action plans was counterproductive for individuals with high levels of habit strength; in contrast, individuals with low levels of habit strength engaged in more exercise after creating action plans. Since then, some researchers have repeated the study with inconsistent results, finding that the correlation between action planning and exercise behavior was not inhibited by habit strength (Di Maio et al., 2021). First, is it superfluous to create action plans for individuals with high habit strength? Second, whether making plans and forming habits is an iterative process that can together facilitate the transformation of intention into behavior. Therefore, the above two issues need to be clarified. Hypothesis 3 considers: *Habit strength moderates the predictive effect of action planning on the exercise behavior of college students.*

It is crucial to investigate the effect of demographic variables on intention-behavior relationships because it may imply inequalities in the social structure of the population (Schüz et al., 2017). The latest study by Rhodes et al. (2022) revised a previous systematic review of potential moderators of intention-behavior relationships in the physical activity domain (Rhodes and Dickau, 2013) and reassessed the moderating role of gender. More than 66% of the studies showed that the gender variable did not affect intention-behavior invariance. The remaining studies concluded that females had stronger intention-behavior relationships than males (Nigg et al., 2009; Xin et al., 2019), or the opposite (Plotnikoff et al., 2012). Will an increasing number of findings in the future support the perspective that “females/males are somehow more devoted to pursuing their own intentions”? Out of curiosity, we propose hypothesis 4: *Gender moderates the predictive effect of exercise intention on the exercise behavior of college students.*

Martin’s (2004) study of student motivation in Australia found that girls were more likely than boys to adopt plans to manage their behavior and to show greater resilience in the face of challenges. To investigate gender differences in the process of health behavior change, Hankonen et al. (2010) conducted a study on people at high risk for type 2 diabetes, trying to test whether changes in action planning could predict changes in exercise behavior. At the baseline level, there was no difference in action planning by gender, but after 3 months, action planning played a more prominent role in exercise behavior change in females than in males. In a study by Magoc et al. (2016), it was found that gender had a moderating effect on the relationship between exercise planning and behavior, with exercise planning being the strongest predictor of exercise behavior among female college students. According to the above findings, there may be gender differences in the field of self-regulation strategy, and action planning may promote exercise behavior in females. Hypothesis 5 is proposed: *Gender moderates the predictive effect of action planning on the exercise behavior of college students.*

**FIGURE 1:**
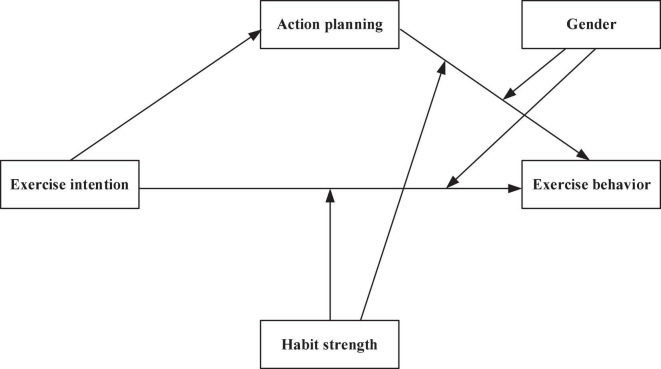
The proposed moderated mediation model.

In summary, the goal of this study is to discuss the mediating mechanism of action planning and the moderating effects of habit strength and gender by analyzing the intention-behavior relationship in the exercise domain. Figure 1 depicts the proposed hypothetical model.

## Materials and methods

### Participants and procedure

A prospective online survey was conducted at two universities in Wuhan, China. Through convenience sampling, the data was collected *via* WeChat groups of 15 sports clubs on campus, which were originally created to advertise in the fall. Specifically, each club leader (i.e., instructor or teacher) assisted us by disseminating the link to the electronic questionnaire to their WeChat group for students to complete. They operated at a more detailed level during the survey, communicating directly with students, but they were not part of the assessment population for this study. At baseline (Time 1; T1), 532 students first provided informed consent, and then filled in the questionnaire assessing exercise intention, action planning, and demographic variables. The follow-up assessment (Time 2; T2) took place 4 weeks later at a club seminar with 489 participants (92% of the initial sample; 43 students lost data), and it included habit strength and exercise behavior. This longitudinal sample’s mean age was 20.61 (SD = 2.10; range 16–29), with 42.54% being male. In terms of educational qualifications, 82% of the longitudinal sample were bachelor level students, and the rest were either master level (14.93%) or Ph.D. students (3.07%). Participants who completed two assessments were rewarded with small gifts.

### Measures

For the purposes of consistency, the questionnaires completed by these college students measured the relevant variables using a distinct definition of exercise. Exercise was defined as a subset of physical activity that was (1) planned, structured, and involved repetitive bodily movements; (2) not a basic daily activity, performed at your own discretion; and (3) intended to improve or maintain the health of one or more body parts (Caspersen et al., 1974; Bouça-Machado et al., 2019).

Three items from the TPB-related structure scale created by Ajzen (2002) were used to assess exercise intention. Specifically, it included “*I plan/intend/hope to exercise for at least 20 minutes, three times per week for the next four weeks.*” Participants evaluated each item on a 7-point Likert-type scale with a minimum value of 1 (completely disagree) and a maximum value of 7 (completely agree). A previous study demonstrated that the Chinese version of the scale had great reliability and validity among college students (Jian et al., 2020). Cronbach’s alpha was 0.91 in this study.

Sniehotta et al. (2005) developed five items to assess action planning, which addressed when, where, who, how long, and how to act. The items were worded: “*For the next four weeks, I have made a detailed plan regarding.*” (1) “*when to exercise*,” (2) “*where to exercise*,” (3) “*how to exercise*,” (4) “*how often to exercise*,” and (5) “*with whom to exercise.*” These items were measured on a 4-point Likert-type scale ranging from 1 (completely disagree) to 4 (completely agree). The original questions were translated into Chinese by two scholars with overseas visiting experience. Cronbach’s alpha was 0.95 in this study.

Gardner et al. (2012) simplified a four-item automaticity subscale (SRBAI) to assess habit strength. The stem “*Exercise is something.*” is followed by “*I do automatically*,” “*I do without having to consciously remember*,” “*I do without thinking*,” and “*I start doing before realizing I am doing it.*” These items were also translated into Chinese by the two scholars mentioned above and scored using the 7-point Likert-type scale. A total score was calculated by adding the scores, which higher values indicate a stronger level of automaticity in exercise. Cronbach’s alpha was 0.89 in this study.

The Physical Activity Rating Scale-3 (PARS-3) was used to assess exercise behavior (Deqing, 1994). The PARS-3 consists of three items that evaluate the amount of exercise done as a result of intensity, duration, and frequency. These items were measured on a 5-point Likert-type scale. The total score of physical activity is calculated as follows: intensity × (duration-1) × frequency, ranging from 0 to 100. The three levels are less than or equal to 19 (light), 20 to 42 (moderate), and more than or equal to 43 (vigorous). Cronbach’s alpha was 0.62 in this study.

### Statistical analyses

Data analyses were carried out using AMOS 23.0 and SPSS 26.0. First, a common method bias test was performed using the unmeasured latent method factor technique based on confirmatory factor analysis (CFA). Second, the differences in exercise intention, action planning, habit strength, and exercise behavior across demographic characteristics were examined using descriptive statistics and independent sample *t*-test. Pearson’s correlation analysis was used to test the correlation coefficients between the continuous variables and whether they were statistically significant. Third, hypothesis testing was performed in three phases, evaluating the mediating role of action planning, the moderating role of habit strength, and the moderating role of gender, in that order. The values of the variables used in all regression models were standard scores (i.e., Z-scores).

## Results

### Common method biases test

A first-order multi-factor oblique intersection measurement model (M1) was constructed using AMOS 23.0. On this basis, a method factor was added to construct the model (M2). Subsequently, the partial fit indices of M1 and M2 were compared. The results showed that ΔRMSEA = 0.015, ΔSRMR = 0.020, ΔTLI = 0.016, and ΔCFI = 0.016. The fluctuations of RMSEA and SRMR did not exceed 0.05, and the fluctuations of TLI and CFI did not exceed 0.1 (Zhonglin et al., 2018), indicating that there was no serious problem of common method bias. For more details on model fit, see the Supplementary material.

### Descriptive statistics and independent sample *t*-test

Descriptive values of absolute and relative frequencies are shown in Table 1. The gender ratio in this sample was balanced, and the majority were undergraduate students. In the past month, 80.16% of them did not participate in offline activities organized by sports clubs, and 60.12% did not achieve a moderate or vigorous amount of exercise.

**TABLE 1:** Frequencies of demographics and amount of exercise.

Variable	Categories	*n*	%
Gender	Male	208	42.54%
	Female	281	57.46%
Educational qualifications	Undergraduate	401	82.00%
	Postgraduate	88	18.00%
Amount of exercise in the past month	Light	294	60.12%
	Moderate	106	21.68%
	Vigorous	89	18.20%
Club offline activities in the past month	Participated	97	19.84%
	Did not participate	392	80.16%

According to the results of the independent samples *t*-test in Table 2, male and female college students had significantly different exercise intention, action planning, habit strength, and exercise behavior (all *p* < 0.001). Male’s scores were higher than those of female. Meanwhile, college students who had participated in offline club activities performed much better on all variables (all *p* < 0.05). The average value of postgraduate students’ exercise behavior was significantly higher than that of undergraduate students (*t* = −3.24, *p* < 0.01).

**TABLE 2:** Statistical differences in different categories of variables.

	EI (M ± SD)	*t*	AP (M ± SD)	*t*	HS (M ± SD)	*t*	EB (M ± SD)	*t*
**Gender**								
Male	16.04 ± 4.04	6.43***	13.53 ± 3.61	5.80***	20.31 ± 4.99	8.76***	30.04 ± 22.91	7.76***
Female	13.49 ± 4.68		11.49 ± 4.13		16.04 ± 5.58		15.32 ± 17.37	
**Educational qualifications**								
Undergraduate	14.45 ± 4.51	–1.29	12.32 ± 4.04	–0.48	17.70 ± 5.66	–1.31	19.93 ± 19.96	−3.24**
Postgraduate	15.15 ± 4.94		12.55 ± 4.04		18.58 ± 6.06		29.10 ± 24.84	
**Club offline activities in the past month**								
Participated	15.58 ± 4.75	2.41*	13.43 ± 4.28	2.95**	20.55 ± 5.19	5.31***	37.29 ± 22.09	8.03***
Did not participate	14.33 ± 4.53		12.09 ± 3.94		17.19 ± 5.67		17.69 ± 19.08	

EI, exercise intention; AP, action planning; HS, habit strength; EB, exercise behavior.

*p < 0.05; **p < 0.01; and ***p < 0.001.

In addition, effect size and statistical power were calculated for the results of independent sample *t*-tests using G*Power 3.1. The Cohen’s d values were higher than 0.5 and the power (1–β) values were higher than 0.8, after pre-setting α = 0.05 and importing the means, standard deviations, and sample size for both the male and female groups. Consequently, we considered the sample to be adequate.

### Correlation analysis

As shown in Table 3, the correlation coefficients of EI, AP, HS, and EB were all statistically significant. The EI was positively correlated with AP (*r* = 0.50, *p* < 0.001), HS (*r* = 0.53, *p* < 0.001), and EB (*r* = 0.42, *p* < 0.001). The AP was positively correlated with HS (*r* = 0.64, *p* < 0.001) and EB (*r* = 0.49, *p* < 0.001). The HS was positively correlated with EB (*r* = 0.51, *p* < 0.001).

**TABLE 3:** Correlations for all variables.

	M ± SD	1	2	3	4
1. Exercise intention	14.58 ± 4.59	–			
2. Action planning	12.36 ± 4.04	0.50***	–		
3. Habit strength	17.85 ± 5.73	0.53***	0.64***	–	
4. Exercise behavior	21.58 ± 21.19	0.42***	0.49***	0.51***	–

***p < 0.001.

### Mediation analysis of action planning

In order to evaluate the mediating role of action planning between exercise intention and exercise behavior among college students while controlling for gender, age, and educational qualifications, Hayes’ (2013) PROCESS macro for SPSS 26.0 was used to select “*Model 4.*” As recommended by Judd and Kenny (1981) and Baron and Kenny (1986), three regression models were constructed by testing the regression coefficients in sequence. First, the results in Table 4 showed that exercise intention significantly influenced exercise behavior in M3 (β = 0.33, *p* < 0.001; the “*c*” in “*Y* = *cX* + *e*_1_” was statistically significant). Second, exercise intention significantly influenced action planning in M4 (β = 0.46, *p* < 0.001), and action planning significantly influenced exercise behavior in M5 (β = 0.35, *p* < 0.001). That is, the “*a*” in “*M* = *aX* + *e*_2_” and the “*b*” in “*Y* = *c′M* + *bM* + *e*_3_” were statistically significant. Third, exercise intention significantly influenced exercise behavior in M5 (β = 0.17, *p* < 0.001; the “*c′*” in “*Y* = *c′M* + *bM* + *e*_3_” was statistically significant). This demonstrated that action planning played a mediating role in the relationship between college students’ exercise intention and exercise behavior. Specifically, the indirect effect of the action planning accounted for 48.52% of the total effect (β = 0.16, *Boot SE* = 0.03, 95% *Boot CI* [0.11, 0.22]).

**TABLE 4:** Results of the test for mediating effects of action planning.

	Outcome variable: Exercise behavior (M3)				Outcome variable: Action planning (M4)				Outcome variable: Exercise behavior (M5)			
	β	*t*	*p*	95% CI	β	*t*	*p*	95% CI	β	*t*	*p*	95% CI
Gender	0.54	6.50	<0.001	[0.38, 0.71]	0.23	2.73	0.007	[0.06, 0.39]	0.46	5.86	<0.001	[0.31, 0.62]
Age	0.00	0.02	0.983	[−0.05, 0.06]	0.05	1.92	0.056	[0.00, 0.11]	−0.02	−0.69	0.494	[−0.07, 0.03]
Educational qualifications	0.44	2.87	0.004	[0.14, 0.75]	−0.21	−1.34	0.182	[−0.51, 0.10]	0.52	3.55	<0.001	[0.23, 0.80]
Exercise intention	0.33	8.10	<0.001	[0.25, 0.41]	0.46	11.29	<0.001	[0.38, 0.54]	0.17	3.96	<0.001	[0.09, 0.26]
Action planning									0.35	8.16	<0.001	[0.27, 0.43]
*R* ^2^	0.26				0.27				0.35			
*F(p)*	42.18 (<0.001)				43.88 (<0.001)				51.65 (<0.001)			

M3, M4, and M5 represent the three regression models constructed sequentially.

The upper and lower limits of the 95% confidence interval do not contain “0” to reach the significant level.

### Moderation analysis of habit strength

Hayes’ (2013) PROCESS macro for SPSS 26.0 was used to select “*Model 15*” to test whether habit strength played a moderating role in the direct and second half paths of the aforementioned mediation model, controlling for gender, age, and educational qualifications. The results in Table 5 showed that the effect of exercise intention × habit strength interaction (Int_1) on exercise behavior was not statistically significant (β = 0.03, *p* > 0.05). Action planning × habit strength interaction (Int_2) significantly influenced exercise behavior in M6 (β = 0.09, *p* < 0.05). Accordingly, habit strength simply moderated the relationship between action planning and exercise behavior while having no influence on the relationship between exercise intention and exercise behavior.

**TABLE 5:** Results of the test for moderating effects of habit strength.

	Outcome variable: Action planning (M4)				Outcome variable: Exercise behavior (M6)			
	β	*t*	*p*	95% CI	β	*t*	*p*	95% CI
Gender	0.23	2.73	0.007	[0.06, 0.39]	0.39	4.94	<0.001	[0.24, 0.55]
Age	0.05	1.92	0.056	[0.00, 0.11]	−0.02	−0.83	0.407	[−0.07, 0.03]
Educational qualifications	−0.21	−1.34	0.182	[−0.51, 0.10]	0.47	3.33	<0.001	[0.19, 0.75]
Exercise intention	0.46	11.29	<0.001	[0.38, 0.54]	0.13	2.95	0.003	[0.04, 0.22]
Action planning					0.26	5.36	<0.001	[0.16, 0.35]
Habit strength					0.21	4.08	<0.001	[0.11, 0.31]
Int_1					0.03	0.83	0.409	[−0.05, 0.11]
Int_2					0.09	2.46	0.014	[0.02, 0.17]
*R* ^2^	0.27				0.39			
*F(p)*	43.88 (<0.001)				37.74 (<0.001)			

Int_1: exercise intention × habit strength; Int_2: action planning × habit strength.

M4 and M6 represent the two regression models constructed sequentially.

**FIGURE 2:**
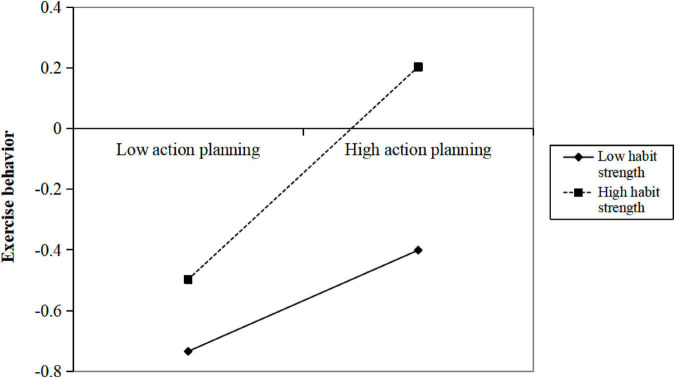
Habit strength moderated the relationship between action planning and exercise behavior. Low: 1 standard deviation below the mean; High: 1 standard deviation above the mean.

A simple slope test was performed using the pick-a-point approach (Aiken and West, 1991) to further analyze the action planning × habit strength interaction in Figure 2. The results showed that a pattern was observed with a stronger relationship between action planning and exercise behavior at high levels (β = 0.35, *p* < 0.001) of habit strength than at medium (β = 0.26, *p* < 0.001) and low levels (β = 0.17, *p* < 0.01). Additionally, as the habit strength level increased, the effect value of the “EI→AP→EB” mediated process tended to gradually rise as well (low HS, β = 0.08, 95% *Boot CI* [0.02, 0.15]; medium HS, β = 0.12, 95% *Boot CI* [0.07, 0.18]; high HS, β = 0.16, 95% *Boot CI* [0.09, 0.24]).

### Moderation analysis of gender

**FIGURE 3:**
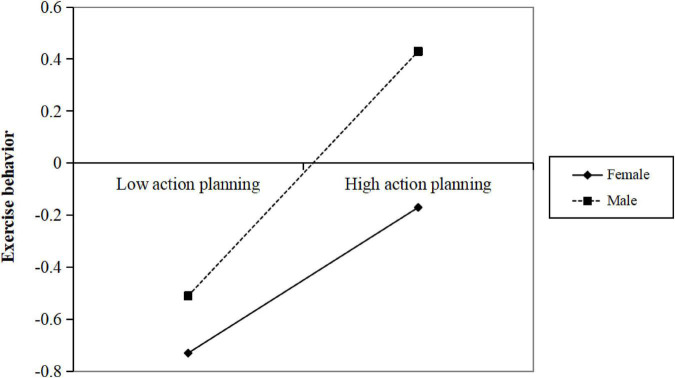
Gender moderated the relationship between action planning and exercise behavior.

Using the PROCESS macro in Hayes (2013) SPSS 26.0, “*Model 15*” was selected to test whether gender played a moderating role in the direct and second half paths of the aforementioned mediation model, controlling for age and education. The results in Table 6 showed that exercise intention × gender interaction (Int_3) had a non-significant influence on exercise behavior (β = 0.16, *p* > 0.05). Action planning × gender interaction (Int_4) significantly influenced exercise behavior in M8 (β = 0.09, *p* < 0.05). Furthermore, in Figure 3, simple slope analysis revealed that the association between action planning and exercise behavior was larger in males than in females.

**TABLE 6:** Results of the test for moderating effects of gender.

	Outcome variable: Action planning (M7)				Outcome variable: Exercise behavior (M8)			
	β	*t*	*p*	95% CI	β	*t*	*p*	95% CI
Age	0.06	2.29	0.023	[0.01, 0.12]	−0.02	−0.62	0.539	[−0.07, 0.04]
Educational qualifications	−0.28	−1.83	0.068	[−0.58, 0.02]	0.48	3.31	0.001	[0.19, 0.76]
Exercise intention	0.49	12.43	<0.001	[0.41, 0.57]	0.12	2.23	0.027	[0.01, 0.23]
Action planning					0.28	5.09	<0.001	[0.17, 0.39]
Gender					0.41	5.22	< 0.001	[0.26, 0.57]
Int_3					0.16	1.80	0.072	[−0.01, 0.33]
Int_4					0.19	2.24	0.026	[0.02, 0.36]
*R* ^2^	0.25				0.37			
*F(p)*	55.28 (<0.001)				39.78 (<0.001)			

Int_3: exercise intention × gender; Int_4: action planning × gender.

M7 and M8 represent the two regression models constructed sequentially.

Since the gender differences exhibited by the sample in the scores of each variable and the stable effect of gender as a control variable on the outcome variable. Two additional testing procedures were used: first, “*Model 17*” was used to test the dual-moderating effects of habit strength and gender; second, “*Model 19*” was used to test three-way interactions. When the moderating effects of habit strength and gender were examined simultaneously, none of the interactions were statistically significant. There was no significant influence on exercise behavior from any of the three-way interactions (i.e., exercise intention × habit strength × gender, and action planning × habit strength × gender). For more details on the regression model, see the Supplementary material.

## Discussion

Our aim in the current study was to examine the mediating role of action planning and the moderating role of habit strength and gender by analyzing the relationship between exercise intention and exercise behavior. Overall, the findings provided support for the idea that physical exercise is determined by a combination of conscious and non-conscious psychological processes.

### The mediating effect of action planning

According to the results of this study, supporting hypothesis 1, action planning partially mediated the relationship between exercise intention and exercise behavior, with an indirect effect of up to 48.52%. The effectiveness of action planning continued to be recognized as one of the most frequently used techniques for bridging the intention-behavior gap (Hagger and Luszczynska, 2014). Specifically, after forming the intention to exercise, the action planning’s function is to transform the “*unconstrained free reaction*” into a “*conscious planning list*,” and thus, bolsters or augments exercise intention with means to promote recall and enactment of the intended behavior.

A study of college students by Fleig et al. (2013) found that intentions facilitated the use of action planning, which in turn promoted exercise. In a young sample from several UK universities, action planning also promoted exercise behavior and partially mediated intention-behavior relationships (Conner et al., 2010). These are consistent with the results of the present study. Looking at past studies on action planning as a mediator, we can see that the above results do not seem to show sufficient novelty. However, it has become an important basis for contributing to the change and maintenance of exercise behavior, the first step on the path from intention to behavior. For example, the so-called volitional stage in the theoretical propositions of the HAPA (Schwarzer, 2008) served as an antecedent variable for coping planning (Reyes Fernández et al., 2015; Wee and Dillon, 2022), action control (Reyes Fernández et al., 2015), and preparatory actions (Barz et al., 2014). Therefore, action planning is adopted with a higher priority among the post-intentional predictors of college students’ exercise behavior.

Theoretically, action planning should be considered in the intention-behavior relationship. On the one hand, it supports the idea that the successful execution of exercise behaviors by college students is partly attributed to a deliberate and rational approach to processing (Rebar et al., 2016), requiring hypothetical reasoning, selection decisions, and self-evaluations (Bagozzi et al., 2003). On the other hand, it is essential to continue exploring the interaction between action planning and other factors (e.g., habits) and transmission mechanisms of action planning with other factors (e.g., preparatory actions). In reality, the action planning includes two advantages that will assist college students in successfully transforming their exercise intentions into exercise behaviors. It has a low response burden, making its promulgation through multiple modes of delivery comparatively easy, and is relatively low-cost (Hagger and Luszczynska, 2014).

### The moderating effect of habit strength

Hypothesis 2 was not supported, and the findings showed that habit strength did not play a moderating role in the relationship between exercise intention and exercise behavior. The result contradicted the view of some dual process models (e.g., Strack and Deutsch, 2004) that suggest conscious processing can be biased by the non-conscious regulatory system through its influence on accessibility to information about behavioral options. Up to now, most of the evidence in the physical activity domain tends to show intention to become less predictive of behavior as habit strength increases (Rhodes and Dickau, 2013; van Bree et al., 2013; Hashim et al., 2014; Gardner, 2015; Rebar et al., 2016). The theoretical argument advanced was that once a behavior becomes habitual, it is less susceptible to intentional control than when it is not habitual. In other words, habitual behaviors rely less on an individual’s subjective consciousness and do not involve a relatively slow and effortful reasoning process.

In contrast to this, a small number of studies found intention to be more predictive of behavior at stronger levels of habit strength. Undergraduate students who reported high habit levels in the vigorous physical activity condition revealed a greater intention-behavior relationship than their moderate and low habit counterparts, according to Rhodes and de Bruijn (2010). de Bruijn et al. (2012) even came up with the result that intention was a stronger predictor of exercise behavior at higher, rather than at lower, levels of exercise habit strength. Apparently, due to the strenuousness and effortfulness of exercise behavior, strong motivational and automatic components may be required simultaneously, rather than a trade-off between these components (Maddux, 1997; Rhodes and de Bruijn, 2010).

Unfortunately, this current study is not supported by either of these empirical evidences. The “habit-intention interaction hypothesis” not only stated that the association between habit and intention as determinants of behavior is like that of a horse race (Adriaanse et al., 2011), but also added that the influence of habit will depend on the degree of familiarity with the focal behavior in a given situation (Triandis, 1977; Gardner et al., 2020). In unfamiliar settings, in which no action traces exist and behaviors are novel, intentions alone will determine behavior. This means that the reason for the insignificant moderating effect of habit strength could be the unstable context in which the college students performed exercise in the past month. The individual’s conscious regulatory processes are dominant in taking action.

When the significant action planning × habit strength interaction was decomposed, findings were also in line with our tentatively formulated hypothesis 3. We found a gradual reinforcement of the predictive effect of action planning on exercise behavior as the level of habit strength increased. It is worth noting that this result opposes the previous general consensus. That is, for individuals with high levels of habit strength for a behavior, developing a plan may disrupt the usual behavior by interfering with the habitual response (Wood and Rünger, 2016). Such as snacking (Adriaanse et al., 2011), recycling behavior (Holland et al., 2006), and smoking (Webb et al., 2009). These studies all found that the stronger an individual’s habit, the less effective their implementation intentions will be. In other words, the plans may only be an effective tool to promote more physical activity in college students with lower levels of habit strength (Maher and Conroy, 2015).

Nevertheless, the present findings underpin that even if college students have a high degree of automaticity in performing exercises, forming a detailed and effective action planning will have a positive effect. Di Maio et al. (2021) likewise supported the view that action planning did not become superfluous, although automatic processes were strong. In habit theory, there are mechanisms by which action plans and habit strength are interrelated and iterative, working together to produce changes in exercise behavior (Lally and Gardner, 2013). After the intention is translated into action, the behavior is about to be repeated, which usually requires continuous motivation (Rothman, 2000) and may also be supported by self-regulatory techniques (Michie et al., 2009). Thus far, the moderating effect of habit on the planning-behavior link has received relatively little attention in physical activity domains, with only two studies by Maher and Conroy (2015) and Di Maio et al. (2021). The future remains to be examined further.

### The moderating effect of gender

In addition to the initially proposed hypotheses 4 and 5, we further analyzed other potential issues regarding gender. Although hypothesis 4 was not supported by the study results, it is consistent with the prevailing theory (Fishbein and Ajzen, 1975). Exercise intention is a powerful predictor of exercise behavior. Most demographic variables followed the principle of invariance in the intention-behavior relationship (Rhodes et al., 2022). The present study, along with a number of large global surveys, had shown differences in physical activity levels between males and females (Hallal et al., 2012; Guthold et al., 2018; de Looze et al., 2019), but this did not serve as evidence to support a gender moderation of intention-behavior relationship. Gender moderated the relationship between action planning and exercise behavior, and hypothesis 5 was supported. Inconsistent with the findings of Hankonen et al. (2010), the association between action planning and exercise behavior was stronger among males, which we assume was due to sample differences. Females between the ages of 50–65 in economically developed areas with a higher risk of type 2 diabetes were driven by a perceived personal health status and were more likely to make a plan and then take action. Conversely, in areas where traditional gender norms prevail, males dominated the advantage of many social resources (de Looze et al., 2019), which was more conducive to going about their plans and doing more physical exercise. Moreover, we found that the moderating role of habit strength in the relationship between action planning and exercise behavior was not interfered with by gender. This means that continuing to develop an action plan can be a universal program for students who have formed positive exercise habits without the need to make deliberate distinctions by gender.

## Limitations and prospectives

The current study adds insights into the intrinsic link between exercise intention, exercise behavior, action planning, and habit strength. In particular, the interaction between action planning and habit strength has a reference value for promoting physical exercise among college students. Undoubtedly, this study has yet to be further improved: First of all, an investigation study in a natural setting cannot provide the same level of power of evidence as a laboratory study, especially with respect to the stability of the control context. This is precisely one of the limitations of the SRHI (Verplanken and Orbell, 2003), which does not capture other components of the habitual experience, such as the dependency on cues and context stability (Hagger, 2018). Follow-up is necessary to clarify whether patterned action, stimulus-response bonding (Grove et al., 2014), and the degree of consistency of cues at the onset of each behavior (Pimm et al., 2016) may bias the results of this study. Secondly, for the sampling procedure, participants who were easily accessible and cooperative were selected for reasons of feasibility of implementing the survey. This may affect the radiometric scope of the findings of this study. Finally, exploring the potential mechanisms of the two manifestations of habit (i.e., habitual instigation and habitual execution; Gardner et al., 2016) in the intention-behavior relationship of exercise, some researchers have suggested that habitual instigation may be more important than habitual execution (Feil et al., 2021). Future studies should pay close attention to all of these.

## Conclusion

Self-reported data from Chinese college students indicates that action planning is a very important predictor of the post-intentional phase and partially mediates the influence of exercise intention on exercise behavior. Habit strength did not play a moderating role in the relationship between exercise intention and exercise behavior. It may be due to an unstable activity context in which no performance history exists and where behaviors are novel, and intentions alone will determine behavior. The predictive effect of action planning on exercise behavior gradually enhances as the level of habitual strength increases, suggesting that for individuals whose exercise has become habitualized, having a plan is not counterproductive and can also promote more exercise.

## Data availability statement

The raw data supporting the conclusions of this article will be made available by the authors, without undue reservation.

## Ethics statement

This study was reviewed and approved by the School of Physical Education at Hubei Business College, Wuhan, China. The benefits and potential risks of participation in this study were explained to participants, and written informed consent was obtained from them.

## Author contributions

LZ: conception, design of the study, and writing. JH: data collection, data organization, and writing. BZ: data analysis. XX: data organization and formatting. JW: revision and finalization of the manuscript. All authors contributed to the article and approved the submitted version.
