# Supplementary material for: The relationship between exercise intention and behavior of Chinese college students: A moderated mediation model
Source: Front Psychol. 2022 Nov 8;13:1006007. doi: 10.3389/fpsyg.2022.1006007 (PMC9679784; doi:10.3389/fpsyg.2022.1006007)
Supplement: Supplementary file 1 [file Table_1.DOCX]

Supplementary Material

# Supplementary Tables

| Model fit results for first-order confirmatory factor analysis | | | | | | | | | | | |
| --- | --- | --- | --- | --- | --- | --- | --- | --- | --- | --- | --- |
|  | *x^2^/df* | AGFI | GFI | NFI | RFI | IFI | TLI | CFI | RMR | SRMR | RMSEA |
| M1 | 3.087 | 0.907 | 0.935 | 0.955 | 0.943 | 0.969 | 0.961 | 0.969 | 0.086 | 0.047 | 0.065 |
| M2 | 2.224 | 0.931 | 0.960 | 0.973 | 0.959 | 0.985 | 0.977 | 0.985 | 0.041 | 0.027 | 0.050 |
| Reference | ＜3 | ＞0.90 | ＞0.90 | ＞0.90 | ＞0.90 | ＞0.90 | ＞0.90 | ＞0.90 | ＜0.05 | ＜0.05 | ＜0.08 |

Results of the test for dual-moderating effects of habit strength and gender

|  | Outcome Variable: | | | | Outcome Variable: | | | |
| --- | --- | --- | --- | --- | --- | --- | --- | --- |
|  | Action planning (M7) | | | | Exercise behavior (M9) | | | |
|  | *β* | *t* | *p* | 95%CI | *β* | *t* | *p* | 95%CI |
| Age | 0.06 | 2.29 | 0.023 | [0.01, 0.12] | -0.02 | -0.81 | 0.421 | [-0.07, 0.03] |
| Educational qualifications | -0.28 | -1.83 | 0.068 | [-0.58, 0.02] | 0.46 | 3.24 | 0.001 | [0.18, 0.74] |
| Exercise intention | 0.49 | 12.43 | < 0.001 | [0.41, 0.57] | 0.09 | 1.53 | 0.127 | [-0.03, 0.21] |
| Action planning |  |  |  |  | 0.21 | 3.28 | 0.001 | [0.08, 0.33] |
| Habit strength |  |  |  |  | 0.21 | 4.11 | < 0.001 | [0.11, 0.31] |
| Int_1 |  |  |  |  | 0.01 | 0.19 | 0.849 | [-0.08, 0.10] |
| Int_2 |  |  |  |  | 0.07 | 1.71 | 0.088 | [-0.01, 0.15] |
| Gender |  |  |  |  | 0.35 | 4.41 | < 0.001 | [0.20, 0.51] |
| Int_3 |  |  |  |  | 0.11 | 1.13 | 0.258 | [-0.08, 0.31] |
| Int_4 |  |  |  |  | 0.14 | 1.50 | 0.135 | [-0.04, 0.33] |
| *R^2^* | 0.25 | | | | 0.39 | | | |
| *F(p)* | 55.28 (< 0.001) | | | | 31.05 (< 0.001) | | | |
| Int_1: exercise intention × habit strength; Int_2: action planning × habit strength; Int_3: exercise intention × gender; Int_4: action planning × gender. | | | | | | | | |
| M7 and M9 represent the two regression models constructed sequentially. | | | | | | | | |

Results of the test for three-way interactions

|  | Outcome Variable: | | | | Outcome Variable: | | | |
| --- | --- | --- | --- | --- | --- | --- | --- | --- |
|  | Action planning (M7) | | | | Exercise behavior (M10) | | | |
|  | *β* | *t* | *p* | 95%CI | *β* | *t* | *p* | 95%CI |
| Age | 0.06 | 2.29 | 0.023 | [0.01, 0.12] | -0.02 | -0.89 | 0.372 | [-0.07, 0.03] |
| Educational qualifications | -0.28 | -1.83 | 0.068 | [-0.58, 0.02] | 0.47 | 3.31 | 0.001 | [0.19, 0.74] |
| Exercise intention | 0.49 | 12.43 | < 0.001 | [0.41, 0.57] | 0.11 | 1.69 | 0.091 | [-0.02, 0.24] |
| Action planning |  |  |  |  | 0.26 | 3.85 | < 0.001 | [0.13, 0.40] |
| Habit strength |  |  |  |  | 0.11 | 1.60 | 0.110 | [-0.02, 0.23] |
| Int_1 |  |  |  |  | 0.01 | 0.08 | 0.935 | [-0.12, 0.13] |
| Int_2 |  |  |  |  | 0.07 | 1.21 | 0.225 | [-0.04, 0.18] |
| Gender |  |  |  |  | 0.35 | 4.04 | < 0.001 | [0.18, 0.52] |
| Int_3 |  |  |  |  | 0.06 | 0.57 | 0.568 | [-0.14, 0.26] |
| Int_4 |  |  |  |  | 0.03 | 0.27 | 0.788 | [-0.18, 0.24] |
| Int_5 |  |  |  |  | 0.28 | 2.60 | 0.010 | [0.07, 0.49] |
| Int_6 |  |  |  |  | -0.03 | -0.34 | 0.731 | [-0.23, 0.16] |
| Int_7 |  |  |  |  | -0.01 | -0.12 | 0.903 | [-0.18, 0.16] |
| *R^2^* | 0.25 | | | | 0.40 | | | |
| *F(p)* | 55.28 (< 0.001) | | | | 24.60 (< 0.001) | | | |
| Int_1: exercise intention × habit strength; Int_2: action planning × habit strength; Int_3: exercise intention × gender; Int_4: action planning × gender; Int_5: habit strength × gender; Int_6: exercise intention × habit strength × gender; Int_7: action planning × habit strength × gender. | | | | | | | | |
| M7 and M10 represent the two regression models constructed sequentially. | | | | | | | | |
